# Supplementary material for: Prevalence and Risk Factors of Gestational Diabetes Mellitus in Bangladesh: Findings from Demographic Health Survey 2017–2018
Source: Int J Environ Res Public Health. 2022 Feb 23;19(5):2583. doi: 10.3390/ijerph19052583 (PMC8909680; doi:10.3390/ijerph19052583)

Table S1: Sociodemographic and pregnancy characteristics of the study participants and prevalence of Gestational Diabetes Mellitus (unweighted frequency)

**Variables**	**Total (unweighted) frequency of pregnant women n (%) N= 265**	**Women with GDM [a] n (%) N=95**	**Women without GDM [a] n (%) N=170**	**P-value**
Age group, n (%)				
<25	151 (57.0)	50 (33.1)	101 (66.9)	0.285
≥ 25	114 (43.0)	45 (39.5)	69 (60.5)	
Education				
Primary or below	103 (38.9)	32 (31.1)	71 (68.9)	0.433
Secondary	121 (45.7)	47 (38.8)	74 (61.2)	
Higher	41 (15.5)	16 (39.0)	25 (61.0)	
Occupation				
Employed	97 (36.6)	28 (28.9)	69 (71.1)	0.072
Unemployed	168 (63.4)	67 (39.9)	101 (60.1)	
Place of residence				
Rural	169 (63.8)	49 (29.0)	120 (71.0)	0.002
Urban	96 (36.2)	46 (52.1)	50 (47.9)	
Wealth index				
Lowest	89 (33.6)	28 (31.5)	61 (68.5)	0.071
Middle	88 (33.2)	27 (30.7)	61 (69.3)	
Highest	88 (33.2)	40 (45.4)	48 (54.5)	
Birth order				0.542
First	89 (33.6)	28 (31.5)	61 (68.5)	0.542
Second	84 (31.7)	33 (39.3)	51 (61.7)	
Third	92 (34.7)	34 (37.0)	58 (63.0)	
Duration of pregnancy in week				
Mean (SD[b])	21.9 (9.6)	18.7 (9.3)	23.6 (9.2)	<0.001
Pregnancy trimesters [c]				<0.001
First	43 (16.8)	30 (69.8)	13 (30.2)	0.000
Second	100 (39.1)	34 (34.0)	66 (66.0)	
Third	113 (44.1)	27 (23.9)	86 (76.1)	
BMI [d]				
Mean (SD[b])	19.6 (4.1)	20.2 (4.3)	19.2 (4.3)	0.072
Hypertension				
Yes	19 (7.2)	9 (47.4)	10 (52.6)	0.277
No	246 (92.8)	86 (35.0)	160 (65.0)	

[a] GDM- Gestational diabetes mellitus.

[b] SD – Standard deviation

[c] Trimesters of pregnancy, first trimester 0-12 weeks, second trimester 13-24 weeks, third trimester > 24 weeks (n=257)

[d] BMI - Pre pregnancy Body mass index (weight in Kg/height in m^2) (n=257), Calculation- subtracted 3 kilograms from current weight for women up to 20 weeks of gestation. Above 20 weeks of gestation, subtracted 3 kilograms plus 0.5 kilograms/week from their current weight.

Figure S1. The Bangladesh Demographic Health Survey (BDHS) 2017-18 sample design and recruitment process [19]

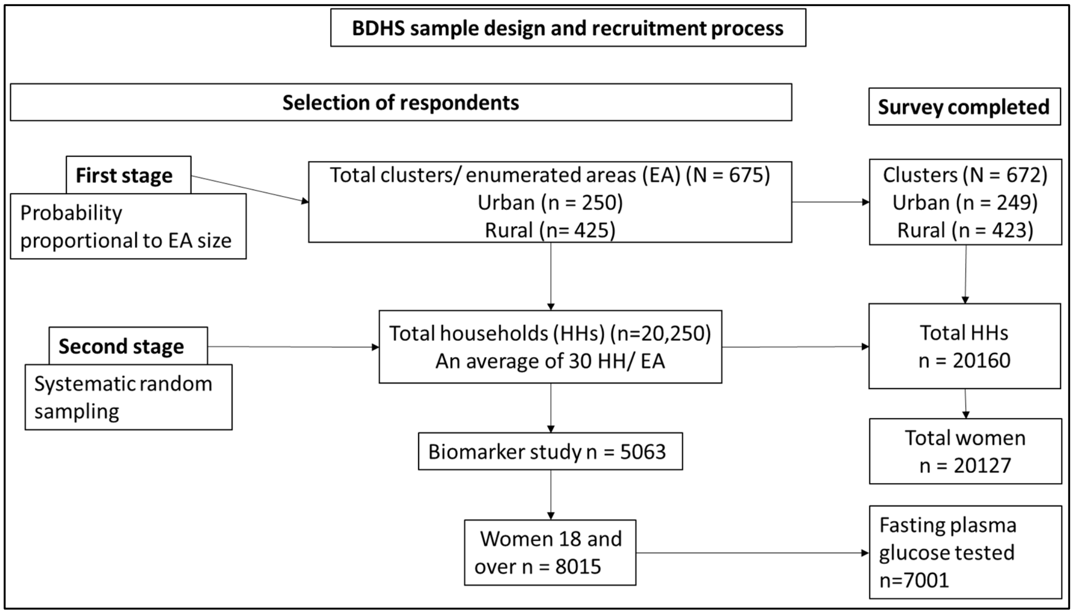

Supplement: Supplementary file 1 [file ijerph-19-02583-s001.zip › ijerph-1556304-supplementary.pdf]
